# Bias and potential premature conclusions regarding the clinical benefits of oXiris in septic adult patients

**DOI:** 10.1186/s13054-023-04594-4

**Published:** 2023-08-11

**Authors:** Jia-Jin Chen, Pei-Chun Lai, Yen-Ta Huang, Chao-Han Lai

**Affiliations:** 1 Department of Nephrology, Chang Gung Memorial Hospital, Linkou Main Branch, Taoyuan City, Taiwan; 2 Education Center, National Cheng Kung University Hospital, College of Medicine, National Cheng Kung University, Tainan, Taiwan; 3 Department of Surgery, National Cheng Kung University Hospital, College of Medicine, National Cheng Kung University, No. 138, Shengli Road, Tainan, 701 Taiwan; 4 Department of Biochemistry and Molecular Biology, College of Medicine, National Cheng Kung University, Tainan, Taiwan; 5 Department of Biostatistics, Vanderbilt University Medical Center, Nashville, TN USA

Dear Editor,

The clinical utilization of oXiris, a groundbreaking filter for continuous kidney replacement therapy featuring an adsorption coating to adsorb endotoxins and remove inflammatory mediators, is increasing among ICU patients with sepsis. It is highly encouraged to validate the impact of oXiris using evidence-based medicine methodologies. We carefully reviewed the recently published systematic review by Wang et al. [1] and found it to be highly intriguing. A significant reduction with a substantial effect in 28-day mortality (odds ratio [OR] 0.53; 95% confidence interval [CI] 0.36–0.77, *I*^2^ = 8%), which is considered one of the critical endpoints, was observed through meta-analysis using data from 7 out of 14 enrolled studies. These 7 studies included two randomized controlled trials (RCTs) and five observational studies. However, the inconsistency between the pooled estimates of RCTs and non-RCTs (OR 1.26 [0.49–3.25], OR 0.44 [0.29–0.67], respectively)] has prompted us to explore additional statistical methods to confirm the certainty of the benefits of oXiris in the outcome of 28-day mortality. Through trial sequential analysis (TSA), Bayesian approach, and limited meta-analysis, we have reservations regarding potential premature conclusions and the presence of publication bias in the analysis.

## Trial sequence analysis

**Fig. 1:**
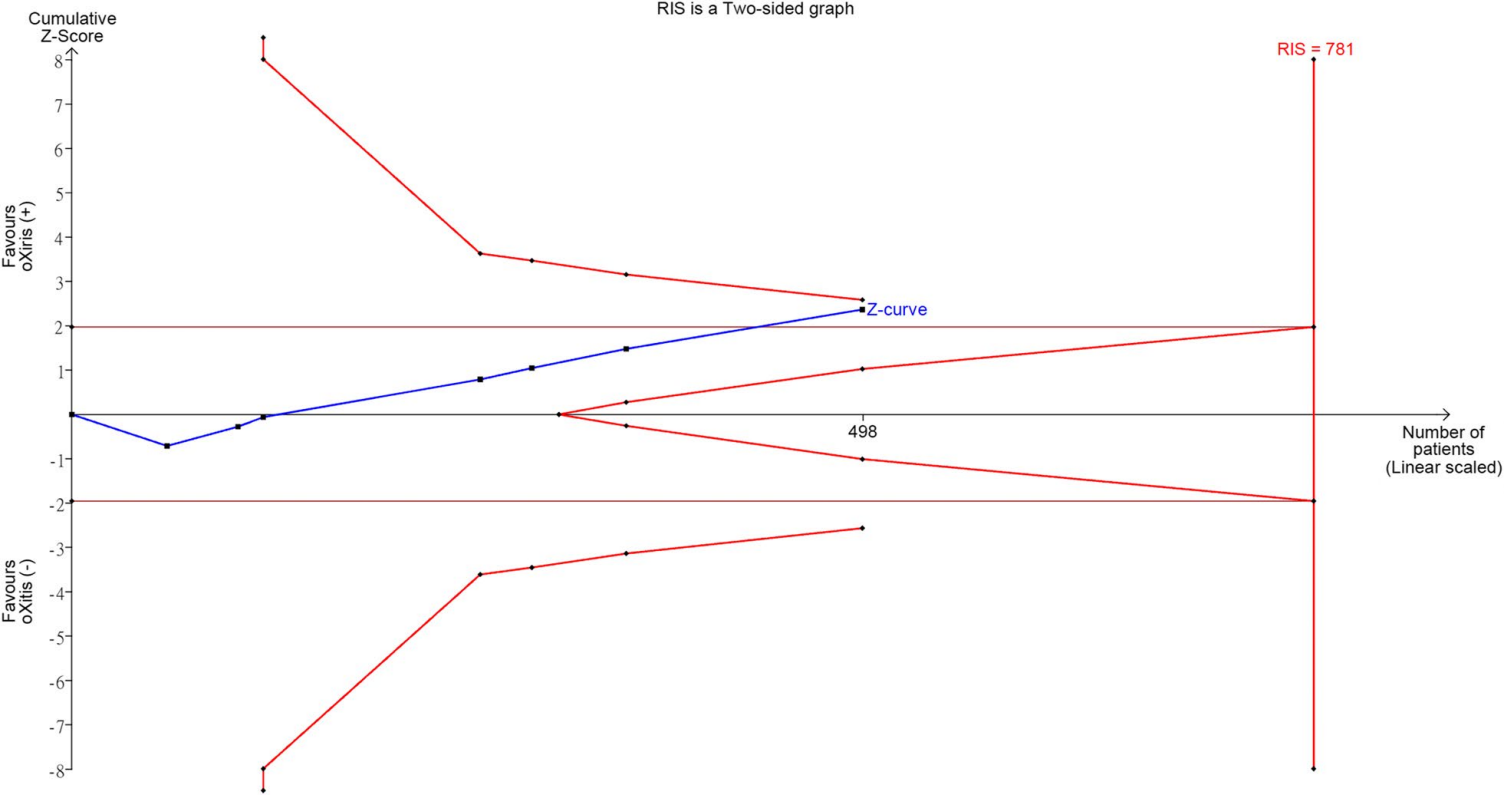
Trial sequential analysis of oXiris for reducing 28-days mortality in comparison with conventional filter

We used TSA software version 0.9.5.10 beta (Copenhagen Trial Unit, Center for Clinical Intervention Research, Rigshospitalet, Copenhagen, Denmark) to assess the potential of false positive results or insufficient sample sizes in determining the effectiveness of oXiris in reducing 28-day mortality, based on the findings from Wang et al. We set the type 1 error at 5% and statistical power at 80%. We used a two-sided boundary type with a penalty of 2 and set the risk reduction ratio at 20%. The random-effect models with the Sidik and Jonkman method were employed, which is recommended by experts, particularly when the number of studies in the meta-analysis is small and when robust evidence is required to draw conclusions [2]. Figure 1 illustrates that the end of cumulative Z-curve extended beyond the conventional test boundary but remained within the upper O'Brien-Fleming monitoring boundary, suggesting the possibility of false positive results. The cumulative sample size was 498, while the required information sample size was 781, indicating that there are insufficient cases to definitively confirm the benefit of oXiris. Consequently, further studies are still necessary.

## Bayesian meta‐analysis

Moreover, we utilized a Bayesian approach as an advanced statistical analysis due to its strong alignment with clinical reasoning. Bayesian analysis has been acknowledged as a practical methodology to interpret clinical trials and formulate clinical practice guidelines [3]. For our analysis, we used Microsoft-Excel-based NetMetaXL V.1.6.1 (Canadian Agency for Drugs and Technologies in Health, Ottawa, Canada) to perform WinBUGS 1.4.3 (MRC Biostatistics Unit, Cambridge, and Imperial College School of Medicine, London, UK) with 10,000 simulations and employed a random-effects model with either vague or informative prior [4]. While a trend of benefit (OR 0.65) was observed, we noted inconclusive ranges in both vague and informative prior analyses (95% credible intervals: 0.31–1.33 and 0.39–1.08, respectively) for the association of lower 28-day mortality. In summary, the results of the Bayesian meta-analysis did not yield robust evidence to support the application of oXiris.

## Limited meta-analysis

It is worth noting that the primary result from Wang et al. [1] heavily relies on retrospective studies, raising concerns about the possibility of publication bias, as negative results from retrospective cohort studies might not have been published. Additionally, half of the enrolled studies (7 out of 14 enrolled trials) did not report the 28-day mortality outcome. Besides, small study effects occur when smaller studies demonstrate different, often larger, treatment effects than larger ones. This phenomenon poses a potential threat to the validity of meta-analyses. To address small study effects, potential publication bias, or non-reporting bias, we performed a limited meta-analysis using the "limitmeta" function in the R metasens package. This extended random-effects model with a shrinkage procedure aimed to account for publication bias [5]. The adjusted OR and 95% CI for oXiris in relation to 28-day mortality was 0.78 and 0.60–1.01, respectively. Again, there is a possibility of non-significance of oXiris treatment in reducing 28-day mortality.

In conclusion, the comprehensive examination undertaken using various methodologies has addressed potential uncertainties and premature conclusions, revealing that biases may lead to different conclusions. We believe that our analysis is essential for *Critical Care* readers to interpret the findings from Wang et al. [1]. However, further rigorous multicountry randomized controlled trials with larger sample sizes are necessary to confirm or refute the potential benefits of oXiris in critical illness septic patients.

## Data Availability

The original data were derived from the manuscript provided by the journal.
